# The anatomical variations of the cubital tunnel in a South African body donor sample

**DOI:** 10.1007/s00276-024-03327-8

**Published:** 2024-03-29

**Authors:** Sophie Rose Munro, Kerryn-Anne Mac Dermott, Kerri Keet

**Affiliations:** 1 Faculty of Medicine and Health Sciences, Stellenbosch University, Cape Town, South Africa; 2 Department of Paediatric Surgery, Surgical Skills Training Centre, Red Cross War Memorial Children’s Hospital, University of Cape Town, Cape Town, South Africa

**Keywords:** Anatomical variation, Anconeus epitrochlearis, Cubital tunnel, Cubital tunnel retinaculum, Cubital tunnel syndrome, Elbow, Ulnar nerve

## Abstract

**Purpose:**

The ulnar nerve (UN) courses through the cubital tunnel, which is a potential site of entrapment. Anatomical variations of the cubital tunnel may contribute towards cubital tunnel syndrome (CuTS), however, these are not well described. The aim was to compare the range of variations and dimensions of the cubital tunnel and the UN between sexes and sides of the body.

**Methods:**

Sixty elbows from 30 embalmed bodies (17 males and 13 females) were dissected. The prevalence of the cubital tunnel retinaculum (CuTR) or *anconeus epitrochlearis* (AE) forming the roof of the tunnel was determined. The length, width, thickness, and diameter of the cubital tunnel and its roof were measured. The diameter of the UN was measured.

**Results:**

The AE was present in 5%, whereas the CuTR was present in the remaining 95% of elbows. The tunnel was 32.1 ± 4.8 mm long, 23.4 ± 14.2 mm wide, 0.18 ± (0.22–0.14) mm thick, and the median diameter was 7.9 ± (9.0–7.1) mm, while the median diameter of the UN was 1.6 ± (1.8–1.3) mm. The AE was thicker than the CuTR (*p* < 0.001) and the UN was larger in elbows with the AE present (*p* = 0.002). The tunnel was longer in males (*p* < 0.001) and wider on the right (*p* = 0.014).

**Conclusion:**

The roof of the cubital tunnel was more frequently composed of the CuTR. The cubital tunnel varied in size between sexes and sides. Future research should investigate the effect of the variations in patients with CuTS.

**Supplementary Information:**

The online version contains supplementary material available at 10.1007/s00276-024-03327-8.

## Introduction

The cubital tunnel is a canal located between the medial epicondyle of the humerus and the olecranon process of the ulnar, through which the ulnar nerve (UN) passes. Typically, the cubital tunnel retinaculum (CuTR) forms the roof of the tunnel and extends between the medial epicondyle and olecranon process [3]. The floor of the tunnel is formed by the anterior, posterior, and oblique bands of the medial collateral ligament (MCL) [29], while the medial epicondyle and olecranon process form the medial and lateral walls, respectively [9]. The CuTR has been described as a fibrous structure formed by the fusion of the antebrachial fascia and the deep fascia of *flexi carpi ulnaris* (FCU) [13], with the retinaculum fibres running perpendicular to that of the aponeurosis of the FCU. In some cases (11.0%), the roof of the tunnel is formed by a variant muscular structure, the *anconeus epitrochlearis* (AE) [16], and more rarely (4.7%), the roof is absent [22]**.** The CuTR and AE both function to retain the UN, which passes underneath the roof structure within the cubital tunnel, before exiting between the two heads of the FCU [22]. The cubital tunnel is, therefore, a potential site of entrapment of the UN [30].

Cubital tunnel syndrome (CuTS) is a neuropathological disorder caused by the entrapment of the UN and is characterised by pain, numbness, and weakness in the hand [2, 9]. The syndrome affects 1.8% of the United States population [2] and is the second most common neuropathy in the upper extremity, following carpal tunnel syndrome [7, 19]. Compression of the UN can be caused by soft tissue structures, including muscles and ligaments, or by bony structures [13] and occurs most commonly at the proximal end of the cubital tunnel [28, 29]. In addition, compression of the UN may result from increased pressure in the cubital tunnel, which is associated with elbow flexion [30]. Although multiple factors contribute to the development of CuTS, the main anatomical structure suggested as being responsible for compressing the UN is the structure forming the roof of the tunnel [23]. However, there is currently no consensus regarding the role of the AE in CuTS. Some authors have described the hypertrophy of AE as the primary cause of CuTS [27, 30], whereas others have argued that the AE is a protective factor against CuTS, due to the reduced rigidity of the muscle compared to the fibrous CuTR [17, 23, 30]. Although Maslow et al. [17] alleged that the AE occupied more space than the CuTR, no measurements were reported. Therefore, when the AE is present, there may be an associated smaller space for the UN to pass through in the cubital tunnel, which could increase the risk of compression of the nerve [17].

Although the structure of the cubital tunnel may contribute towards the development of CuTS [23], research describing the anatomical variations and dimensions of the cubital tunnel is limited. Few studies have reported the dimensions (length, width, thickness, and diameter) of the cubital tunnel and the diameter of the UN in the tunnel. Knowledge of the range of dimensions may be useful in furthering the understanding of UN entrapment at the elbow and may advise orthopaedic surgeons in cubital tunnel decompression procedures. Thus, this study aimed to determine the prevalence of the CuTR or the AE forming the roof of the cubital tunnel. This study also compared the dimensions of the cubital tunnel and the UN between the sexes and sides of the body and determined the relationship between the diameters of the cubital tunnel and the UN.

## Methods and materials

### Study sampling

This cross-sectional dissection-based descriptive study was conducted over ten months (February 2023–November 2023) at a tertiary institution in South Africa. The inclusion criteria included body donors aged 18 years or older and of male or female biological sex. Bodies were excluded if the elbows were fixed at an angle of 90° or less, and if the presence of pathologies obstructed or distorted the structures in both of the elbows. The Anatomical Quality Assurance (AQUA) checklist was followed (Online Resource 1). The study was conducted in accordance with the ethical principles and guidelines set out by the World Medical Association Declaration of Helsinki for Medical Research Involving Human Subjects [6]. Bodies were sourced in accordance with the National Health Act 61 of 2003 of South Africa [25]. Ethical approval was granted by the institution's Undergraduate Research Ethics Committee.

### Dissection and identification of the cubital tunnel

A modified dissection protocol utilising the Gray’s Clinical Photographic Dissector of the Human Body was followed to expose the cubital tunnel and the UN, following dissection by undergraduate health sciences students [15]. The bodies were placed in the prone position with the elbows extended. The subcutaneous fat and connective tissue were carefully removed to maintain the structural integrity of the roof of the cubital tunnel. The UN was identified proximal to the opening of the tunnel and cleaned of connective tissue and fascia. The CuTR contains transverse fibres that originate from the olecranon process and insert into the medial epicondyle. This fibre direction was used to identify the proximal and distal ends of the tunnel and to identify the CuTR. The proximal opening of the tunnel was identified as the location of entry of the UN, whereas the distal end of the tunnel was demarcated as the point where the muscle fibres of the FCU blend into the fibres of the CuTR.

## Data collection

**Fig. 1:**
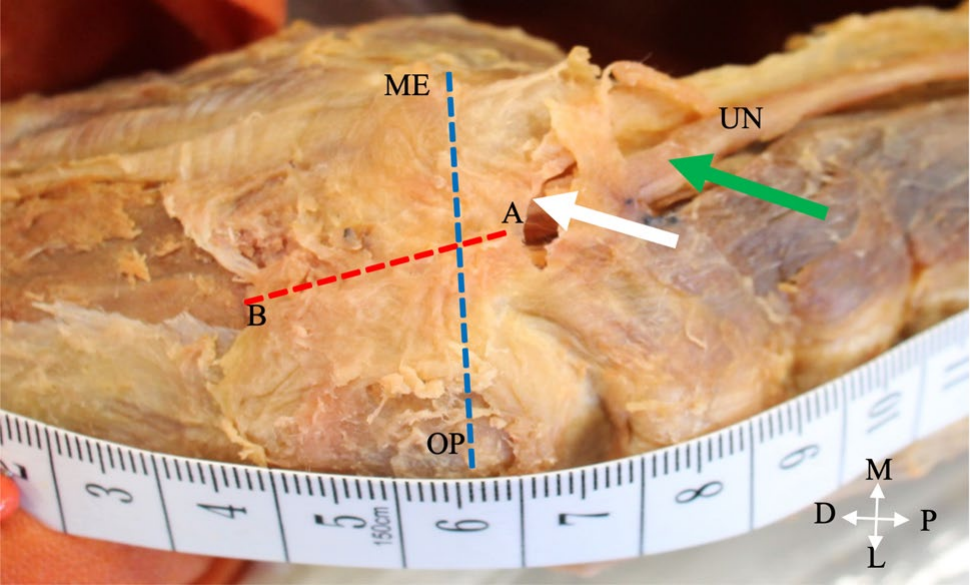
The measurement points of the cubital tunnel retinaculum and ulnar nerve on a right elbow (posterior view). Blue line, length of CuTR; red line, width of CuTR; white arrow, thickness of the CuTR; green arrow, diameter of ulnar nerve; A proximal entry point of the UN; B distal exit point of the UN; D distal; L lateral; M medial, ME medial epicondyle; OP olecranon process; P proximal; UN ulnar nerve

**Fig. 2:**
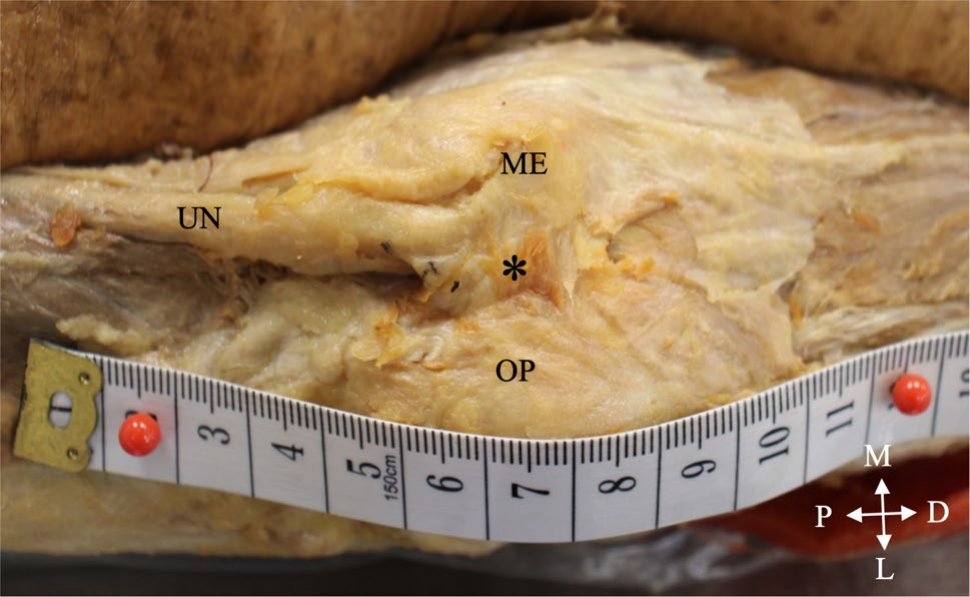
The anconeus epitrochlearis (*) forming the roof of the cubital tunnel in a left elbow (posterior view). D distal; L lateral; M medial; ME medial epicondyle; OP olecranon process; P proximal; UN ulnar nerve

The structure forming the roof of the tunnel was identified as either the CuTR or the AE. The length of this structure was measured in a straight line (Fig. 1, blue line) by securing a piece of non-stretchable ribbon from the origin at the medial epicondyle to the insertion point at the olecranon process with two pins. The ribbon and pins were removed together and placed taunt onto a polystyrene box and the distance between the two pins was measured along the ribbon with a digital calliper (Origin (0–150 mm)) (Online Resource 2, Fig. s1). The width of the roof structure was measured from the proximal entry point of the UN into the tunnel (Fig. 1, point A) to the distal exit point (Fig. 1, point B) where the fibres of the roof blended with the fibres of the forearm muscles, using the same ribbon and pins method as described above. The thickness of the roof structure (Fig. 1, white arrow) was measured directly with the calliper at the proximal entry point of the UN and the diameter of the tunnel (Online Resource 2, Fig. s2, green line) was measured by placing the ribbon on the floor of the tunnel, securing it with a pin, pulling it up and marking the location of the roof with a black marker. The ribbon was subsequently removed and placed on the polystyrene box in a taunt position and the distance between the pin and the marker was measured. The UN was lifted with forceps, thus freeing it from the heads of the *triceps brachii* muscle, and the transverse diameter was measured at the proximal entry point of the tunnel (Fig. 1, green arrow). The diameter of the UN was used to calculate the cross-sectional area (CSA) using the equation CSA = πr^2^ (the diameter was halved to determine the radius (r)). Each of the structures was measured three times and the average thereof was used in data analysis Fig. 2. Photographs were taken with a Canon EOS 2000D camera.

### Data analysis

The Statistical Package for Social Sciences (SPSS) (version 29) was used in consultation with a biostatistician. Intra- and inter-observer reliability tests were conducted on 10% of the sample and analysed with Bland–Altman plots. Fisher exact tests were used to compare the prevalence of AE and CuTR between the sexes and sides of the body. Shapiro–Wilk tests were utilised to determine whether the measurements were normally distributed. For measurements that were normally distributed, independent *t-tests* were used to compare the differences between the sexes, while differences between the sides of the body were compared with paired *t-tests*. Non-parametric tests were used for measurements that were not normally distributed, namely the Mann–Whitney *U-tests* for differences between sexes and the related samples Wilcoxon signed-rank tests for the sides of the body. Finally, a Pearson correlation test was conducted on the diameters of the UN and the cubital tunnel. A *p-value* < 0.05 was deemed significant.

## Results

### Sample

Of the forty (*N* = 40) bodies available for inclusion in the study, nine had damaged cubital tunnels and UNs from previous dissections in both elbows and one male had an elbow fixed at an angle less than 90°. Therefore, 30 bodies (60 elbows) were included in the final sample, of which 13 (43.3%) were female and 17 (56.7%) were male. As a result of previous dissection, not all measurements were able to be taken in the full sample. The length of the structure forming the roof (CuTR or AE) was measured in every elbow, however, the width, thickness, diameter of the tunnel, and diameter of the UN were not measured in some cases (Table 1).

**Table 1:** Sample size of measurements

	Length of roof structure	Width of roof structure	Thickness of roof structure	Diameter of the tunnel	Diameter of the ulnar nerve
Sample Size (number)	60	48	49	48	59

### Prevalence of anconeus epitrochlearis

**Fig. 3:**
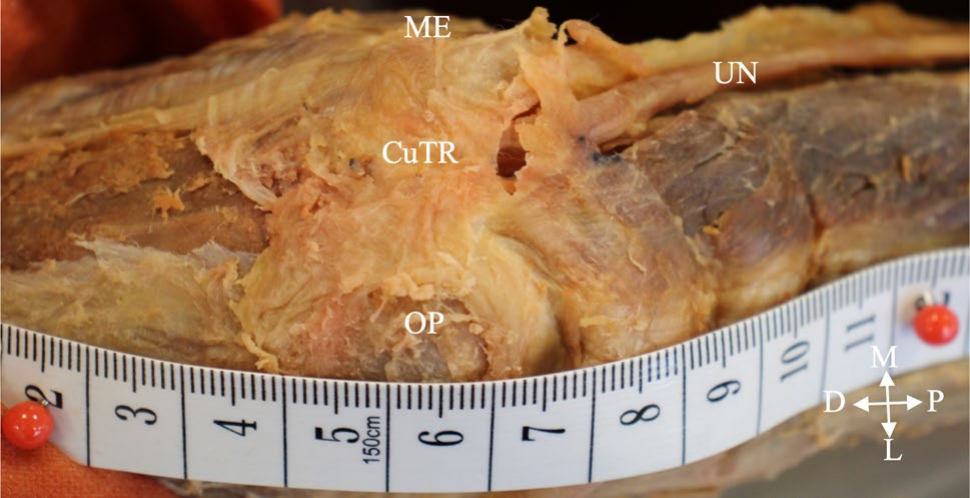
The cubital tunnel retinaculum (CuTR) forming the roof of the cubital tunnel in a right elbow (posterior view). CuTR cubital tunnel retinaculum; D distal; L lateral; M medial; ME medial epicondyle; OP olecranon process; P proximal; UN ulnar nerve

The AE formed the roof of the cubital tunnel in three (5%) unilateral elbows (Fig. 2), while the remaining 57 (95%) elbows all contained the CuTR (Fig. 3). The AE was present in two males on the left and right sides respectively, and in one female on the left side. There were no significant differences in the prevalence of AE or CuTR between sexes and sides (*p* = 1.0) (Table 2).

**Table 2:** Prevalence of cubital tunnel retinaculum and anconeus epitrochlearis between sexes and sides

	Sex: Number (%)		Total: Number (%)	Sides: Number (%)		Total: Number (%)
	Female	Male		Left	Right	
CuTR	25 (41.7)	32 (53.3)	57 (95.0)	28 (46.7)	29 (48.3)	57 (95.0)
AE	1 (1.7)	2 (3.3)	3 (5.0)	2 (3.3)	1 (1.7)	3 (5.0)
*p-value*	1.0			1.0		

Key: AE: *anconeus epitrochlearis;* CuTR: cubital tunnel retinaculum; %: percentage

### Measurements of the *anconeus epitrochlearis* and cubital tunnel retinaculum

The length and width of the roof structure were normally distributed, while the thickness and diameter of the tunnel, and the diameter and the CSA of the UN were not normally distributed. The mean lengths of the CuTR and AE were 32.2 ± 4.9 mm and 31.2 ± 1.7 mm, respectively (Table 3), which were not significantly different (*p* = 0.717). Although the width of the AE was smaller than that of the CuTR (16.4 ± 0.4 mm vs 23.8 ± 4.9 mm), there was no significant difference between these two widths (*p* = 0.12). The AE was, however, significantly thicker than the CuTR (*p* < 0.001; 0.70 ± 0.58 mm vs 0.18 ± 0.08 mm). There was no significant difference in the diameter of the tunnel between elbows with CuTR (8.2 ± 2.0 mm) and those with AE (7.1 ± 1.6 mm) (*p* = 0.362). However, the UN had a significantly larger diameter when the AE was present (2.5 ± 0.9 mm) compared to when CuTR was present (1.6 ± 0.5 mm) (*p* = 0.002). Thus, the CSA of the UN was larger in elbows with an AE (5.3 ± 3.4mm^2^) compared to those with the CuTR (2.1 ± 1.4mm^2^) (*p* < 0.001).

**Table 3:** Measurements of the cubital tunnel and the ulnar nerve according to the structure forming the roof (cubital tunnel retinaculum or anconeus

	Dimensions: Mean ± Standard Deviation					
	Length (mm)	Width (mm)	Thickness (mm)	Diameter of tunnel (mm)	Diameter of UN (mm)	CSA of UN (mm^2^)
CuTR	32.2 ± 4.9	23.8 ± 4.9	0.18 ± 0.08	8.2 ± 2.0	1.6 ± 0.5	2.1 ± 1.4
AE	31.2 ± 1.7	16.4 ± 0.4	0.70 ± 0.58	7.1 ± 1.6	2.5 ± 0.9	5.3 ± 3.4
*p-value*	0.717	0.12	** < 0.001**	0.362	**0.002**	** < 0.001**

Key: AE: *anconeus epitrochlearis;,* CuTR: cubital tunnel retinaculum; CSA: cross sectional area; UN: ulnar nerve

Bold values indicate statistical significance (*p* < 0.05) *epitrochlearis*)

### Comparison of the measurements of the roof structure between sides and sexes

As the length and width of the CuTR and AE were not significantly different, the measurements were pooled for the comparison between sexes and sides. The length (*n* = 60), width (*n* = 48), and thickness (*n* = 49) of the roof and the diameter (*n* = 48) of the cubital tunnel were compared between sexes. Only bilateral measurements were included when sides of the body were compared: length (*n* = 60); width (*n* = 36), thickness (*n* = 38) and diameter (*n* = 36). The mean length of the roof structure was 32.1 ± 4.8 mm. No difference in the mean length was found between the left and right sides (*p* = 0.422), however, males had a longer roof structure than females (*p* < 0.001; Tables 4, 5). The mean width of the roof structure was 23.4 ± 14.1 mm which was significantly wider on the right (24.4 ± 5.5 mm) than on the left side (21.4 ± 4.1 mm) (*p* = 0.014). However, there was no significant difference (*p* = 0.856) in the width of the roof between males and females. The median and interquartile range (IQR) of the thickness of the roof structure was 0.18 ± (0.22 – 0.14) mm. There was no significant difference (*p* = 0.094) in the thickness of the roof structure between the left and right sides. Moreover, there was no significant difference (*p* = 0.644) in the thickness between the sexes. The median diameter of the tunnel was 7.9 ± (9.0–7.1) mm., and similarly, there was no significant difference between the sides of the body (*p* = 0.372) and between the sexes (*p* = 0.153) (Tables 4, 5) (Online Resource 3, Figs. s3, s4, s5, s6).

**Table 4:** Measurements of the cubital tunnel according to sides

	Mean ± Standard Deviation (range)		Median ± (Inter Quartile Range*) (range)	
Sides	Length (mm)	Width (mm)	Thickness (mm)	Diameter (mm)
Left	32.5 ± 4.9 (21.6 – 44.1)	21.4 ± 4.1 (14.15 – 28.48)	0.20 ± (0.23 – 0.17) (0.11 – 1.37)	7.9 ± (8.8 – 6.8) (4.5 – 14.4)
Right	31.8 ± 4.7 (20.2 – 41.4)	24. 4 ± 5.5 (14.7 – 34.0)	0.18 ± (0.21 – 0.12) (0.08 – 0.42)	7.8 ± (9.1 – 7.8) (5.1 – 12.5)
*p-value*	0.422	**0.014**	0.094	0.372
Both Sides	32.1 ± 4.8 (20.2 – 44.1)	23.4 ± 14.2 (14.2 – 34.0)	0.18 ± (0.22 – 0.14) (0.08 – 1.37)	7.9 ± (9.0 – 7.1) (4.6 – 14.4)

^*^IQR is reported as (Q3 – Q1)

Bold values indicate statistical significance (*p* < 0.05)

**Table 5:** Measurements of the cubital tunnel according to sex

	Mean ± Standard Deviation (range)		Median ± (Inter Quartile Range*) (range)	
Sex	Length (mm)	Width (mm)	Thickness (mm)	Diameter (mm)
Male	34.1 ± 4.1 (27.5 – 44.1)	23.2 ± 5.5 (14.15 – 34.01)	0.18 ± (0.23 – 0.13) (0.09 – 0.50)	8.1 ± (9.4 – 7.3) (4.6 – 12.9)
Female	29.6 ± 4.6 (20.2 – 38.8	22.9 ± 4.2 (15.3 – 32.5)	0.18 ± (0.22 – 0.16) (0.08 – 1.37)	7.4 ± (8.5 – 6.9) (5.7 – 14.4)
*p-value*	** < 0.001**	0.856	0.644	0.153
Both Sexes	32.1 ± 4.8 (20.2 – 44.1)	23.4 ± 14.2 (14.2 – 34.0)	0.18 ± (0.22 – 0.14) (0.08 – 1.37)	7.9 ± (9.0 – 7.1) (4.6 – 14.4)

Key: *IQR is reported as (Q3 – Q1)

Bold values indicate statistical significance (*p* < 0.05)

### Measurement of the ulnar nerve

The UN diameter (*p* < 0.001) (*n* = 59) and consequently the CSA (*p* < 0.001) were not normally distributed. The median UN diameter was 1.5 ± (1.8 – 1.3) mm and the median CSA was 1.8 ± (2.5–1.2) mm^2^ (Tables 6, 7). No significant difference was found in the UN diameter between the sexes (*p* = 0.328) or sides (*p* = 0.226). (Online Resource 3, Fig. 7). Consequently, there were no significant differences in the CSA between the sexes; (*p* = 0.264) and sides (*p* = 0.336). The diameters of the tunnel (*n* = 48) and the UN (*n* = 59) were compared in 48 elbows. There was no statistically significant correlation between the diameter of the tunnel and the diameter of the UN (R^2^ = 0.022; *p* = 0.131) (Online Resource 3, Fig. 8), including when the outliers and the measurements from elbows in which the AE was present were excluded.

**Table 6:** Diameter and cross-sectional area of the ulnar nerve according to sides

	Median ± (Inter Quartile Range*) (range)	
Side	Diameter of UN (mm)	CSA of UN (mm^2^)
Left	1.4 ± (1.8 – 1.2) (0.3 – 3.4)	1.6 ± (2.6 – 1.2) (0.1 – 9.1)
Right	1.6 ± (1.8 – 1.3) (1.0 – 3.4)	1.8 ± (2.4 – 1.5) (0.7 – 5.7)
*p-value*	0.226	0.336
Both Sides	1.5 ± (1.8 – 1.3) (0.3 – 3.4)	1.8 ± (2.5 – 1.2) (0.1 – 9.1)

Key: *IQR is reported as (Q3 – Q1); CSA: Cross-sectional area, UN: ulnar nerve

**Table 7:** Measurement of the ulnar nerve according to sex

	Median ± (Inter Quartile Range*) (range)	
Sex	Diameter of UN (mm)	CSA of UN (mm^2^)
Male	1.6 ± (1.9 – 1.2) (0.3 – 3.4)	1.6 ± (2.8 – 1.2) (0.1 – 9.1)
Female	1.5 ± (1.8 – 1.3) (1.0– 2.5)	1.5 ± (2.4 – 1.1) (1.0 – 2.5)
*p-value*	0.328	0.264
Both Sexes	1.5 ± (1.8 – 1.3) (0.3– 3.4)	1.8 ± (2.5 – 1.2) (0.1 – 9.1)

Key: *IQR is reported as (Q3 – Q1); CSA: Cross-sectional area, UN: ulnar nerve

## Reliability

Six elbows were included for the intra- and inter-observer reliability for each measurement. There was a strong consensus between the original measurements and the reliability measures found by the Bland–Altman intra- and inter-observer reliability tests, indicating the methodology is reliable and reproducible with low error (Online Resource 4).

## Discussion

This study assessed the variations and measurements of the cubital tunnel and the UN and found that the roof of the tunnel can be composed of two different structures: the AE or, more prevalently, the CuTR. The structure forming the roof was longer in males than in females, while the right elbows had wider roof structures. In cases where the AE was present, the AE was thicker than the CuTR. Interestingly, the diameter and CSA of the UN were larger when the AE was present. The AE muscle was present in 5% of this sample, which is consistent with previous studies in which the prevalence ranged from 5.4 to 13.3% [10, 21, 23, 27, 29] (Table 8). The prevalence of the AE in males ranged from 1.8 to 8.7% [21, 27], and therefore the prevalence found in the present study (3.3%) falls within this range. The prevalence of AE was lower in females (1.7%) than in males and was lower than that reported in other studies (4.5–10%) [10, 27]. The lower prevalence of AE in the present study could be attributed to the lower sample size of females (*n* = 13) compared to males (*n* = 17). When compared to the dissection study of Suwannakhan et al. [27], the prevalence of AE in the right and left elbows in the current study was similar. Suwannakhan et al. [27] reported a 3.6% prevalence of AE in left elbows and 2.7% in right elbows, similarly, the current study found a higher prevalence of AE in left elbows than right with a 3.3% and 1.7% prevalence in left and right elbows respectively. In contrast to the present study in which no bilateral presence of the AE was found, Suwannakhan et al. [27] reported one case of the bilateral presence of the AE in one female.

**Table 8:** Prevalence of *anconeus epitrochlearis* (AE) in previous studies

Author [reference] (date)	Sample Size (number of limbs)	Study Type	Prevalence of AE				
			Total (%)	Male (%)	Female (%)	Right (%)	Left (%)
Wilson et al. [29] (2016)	168	Surgical Investigation	5.4	NR	NR	NR	NR
Nascimento et al. [21] (2018)	218	Imaging (MRI)^b^	13.3	8.7	4.6	NR	NR
Park et al*.* [23] (2018)	142	Surgical Investigation	8.5	NR	NR	NR	NR
Grewal et al*.*[10] (2018)	40	Cadaveric Dissection ^b^	12.5	2.5	10	NR	NR
Suwannakhan et al*.*[27] (2020)	112	Cadaveric Dissection ^b^	6.3	1.8	4.5	2.7	3.6
Current Study (2023)	60	Cadaveric Dissection	5.0	1.7	3.3	1.7	3.3

Key: AE: *anconeus epitrochlearis*; NR: Not reported; b asymptotic patients; MRI: magnetic resonance imaging; %: percentage

The thickness of the AE (0.70 ± 0.58 mm) was greater than the thickness of the CuTR (0.18 ± 0.08 mm) in this study, which supports the suggestion by Maslow et al. [17] that AE occupies more space than the CuTR. In contrast, the AE was reported by Grewal et al. [10] as 7 mm (range 2–13 mm) thick. The difference in measurement could be due to differences in study designs as Grewal’s study included patients with CuTS and were measured from magnetic resonance imaging (MRI) images. The present study found no significant differences in the thickness of the roof structure between sexes and sides of the body, but to the best of the authors’ knowledge, no other study has compared the thickness of the roof structure between sexes and sides. Males had a longer structure forming the roof of the cubital tunnel than females in the present study, which may be attributed to sexual dimorphism. Few previous studies have investigated the length of the CuTR. However, Grewal et al. [10] reported the mean length of the AE as 22.0 mm (range 15.0 – 32.0 mm) from MRI images and 35.0 mm (range 30.0 – 43.0 mm) using a microcaliper in their dissection study, which is similar to the length found in the current study (31.2 ± 1.7 mm). There was no significant difference between the UN diameter and CSA between sex and sides. Letissier et al. [14], reported that males had a larger UN CSA than females, although no significant differences were found between the left and right sides. Interestingly, the diameter, and therefore, the CSA of the ulnar nerve was significantly larger in elbows with an AE present in this study.

There were no significant differences in the diameter of the cubital tunnel between the sexes and sides of the body. In addition, no significant correlation between the UN diameter and the cubital tunnel diameter was found in this study, although James and Sutton [11] reported a linear relationship between the diameter of the tunnel and the CSA of the UN. However, these measurements were taken when the elbow was flexed at a 90-degree angle compared to the present study where the elbows were extended at an angle larger than 90 degrees [11], which may explain the difference in findings.

The AE is suggested to be a variant muscle in humans, with the function of the muscle not clearly defined [21]. There is no consensus on the potential role that AE plays in the development of CuTS. One study suggests that the presence of the AE may reduce the risk of the development of CuTS due to its decreased rigidity at the entry point of the UN into the tunnel [29]. Wilson et al. [29] reported that the AE was present significantly less frequently in patients with CuTS than in those who did not have CuTS, and thus concluded that AE is a “protective factor” in ulnar neuropathy. In contrast, due to the muscle’s hypertrophic ability, the AE may be a contributing factor in the development of CuTS [29]. In patients in which the AE was present, CuTS developed in the dominant arm more frequently (88.9%) than in those who had CuTS with a CuTR forming the roof of the tunnel [29]. Wilson et al*.* [29] therefore, proposed that hypertrophy of the AE muscle, secondary to extensive use of the dominant arm, contributes towards CuTS. Another study found four cases of UN neuropathy that were secondary to AE hypertrophy as a result of increased elbow flexion [18]. This statement is supported by a third study that reported the presence of hypertrophied AE in three baseball players who developed CuTS [14].

The length of the cubital tunnel roof increases with an increasing degree of elbow flexion, suggesting the roof structure becomes more taut when the elbow is increasingly flexed [11]. This is an important clinical consideration for UN decompression surgery as along with the length increase with flexion, the area of the tunnel also increases. An increased cubital tunnel area provides a larger, safer, surgical field for instruments [11]. The width of the cubital tunnel is clinically relevant as it ultimately describes the length of the portion of the UN that courses through the tunnel. In UN decompression surgery, the structure overlying the UN is cut to prohibit the compression of the nerve [26]. The present study found that the tunnel was wider on the right side of individuals than on the left side. The majority of the population has a right-handed dominance (88–90%) compared to left-handed (10–12%) [12]. The larger width of the tunnel roof structure in the right elbows may be further linked to increased use of the dominant arm.

There were some limitations of the study. Firstly, the number of males and females was not equal due to the limited sample size. The body donors were pre-dissected by medical students, and thus, in some of the individuals, several structures of interest were damaged and therefore the full range of measurements could not be taken. As the cadavers were formalin-fixed, the range of motion of the elbow joints was limited and, although care was taken to ensure consistency, the arm may not have been in the same position in all of the bodies while measurements were taken. The effect of variation of the elbow flexion angle on the dimensions of the tunnel could not be investigated due to the formalin fixation. No medical history was available for the body donors and it was therefore unknown whether any suffered from CuTS. Care must also be taken when interpreting the results of the CSA of the UN as the formula used assumed the nerve was round, however, the nerve may not have necessarily been round in every elbow. Lastly, the difference in reported measurements of the cubital tunnel could be attributed to the effect of ageing on both the AE [20] and the retinaculum [8], however, the age distribution in the sample was unknown and thus could not be taken into consideration.

The presence of ossification in the cubital tunnel was reported as a cause of UN entrapment in a recent case report by Vojtêch et al. [28]. The presence of an accessory ossicle in the cubital tunnel may be congenital or result from pathology and trauma. As the present study focused on the soft tissue of the cubital tunnel, the bony features were not observed. However, no obvious accessory ossicles were detected. Future anatomical studies should document the prevalence of an accessory ossicle in the cubital tunnel and this rare aetiology of cubital tunnel syndrome should be further investigated in clinical studies.

## Conclusion

This study confirms previous research on the low prevalence of the AE forming the roof of the cubital tunnel (5%). The thickness of the AE and the UN diameter and CSA were also significantly larger than when the more prevalent CuTR was observed. This was the first study to compare the thickness of the roof structure with sexes and sides. The roof structure was longer in males and wider in right elbows. There was no significant relationship between the diameter of the tunnel and the diameter of the UN. Future studies could investigate the effect that the flexion angle of the elbow has on the dimensions of the cubital tunnel to provide clinicians with a more comprehensive description of the cubital tunnel for performing UN decompression and cubital tunnel release surgeries. This study provides detailed anatomical information on the variations and measurements of the cubital tunnel and the UN, which may assist in the further understanding of the aetiology of CuTS in a South African setting.

### Supplementary Information

Below is the link to the electronic supplementary material.

Supplementary file1 (DOCX 2964 kb)

## Data Availability

Not applicable.
